# Supplementary material for: Saccharomyces boulardii (CNCM I-745) improves intestinal damage in sepsis by remodeling intestinal flora structure
Source: Front Cell Infect Microbiol. 2025 Jul 31;15:1602792. doi: 10.3389/fcimb.2025.1602792 (PMC12350329; doi:10.3389/fcimb.2025.1602792)
Supplement: Supplementary file 1 [file DataSheet1.zip › summary/summary/6_Taxonomy_community/1_Taxonomy_profiling/6_Genus/Group/Genus_abund_top30_Group.heatmap.pdf]

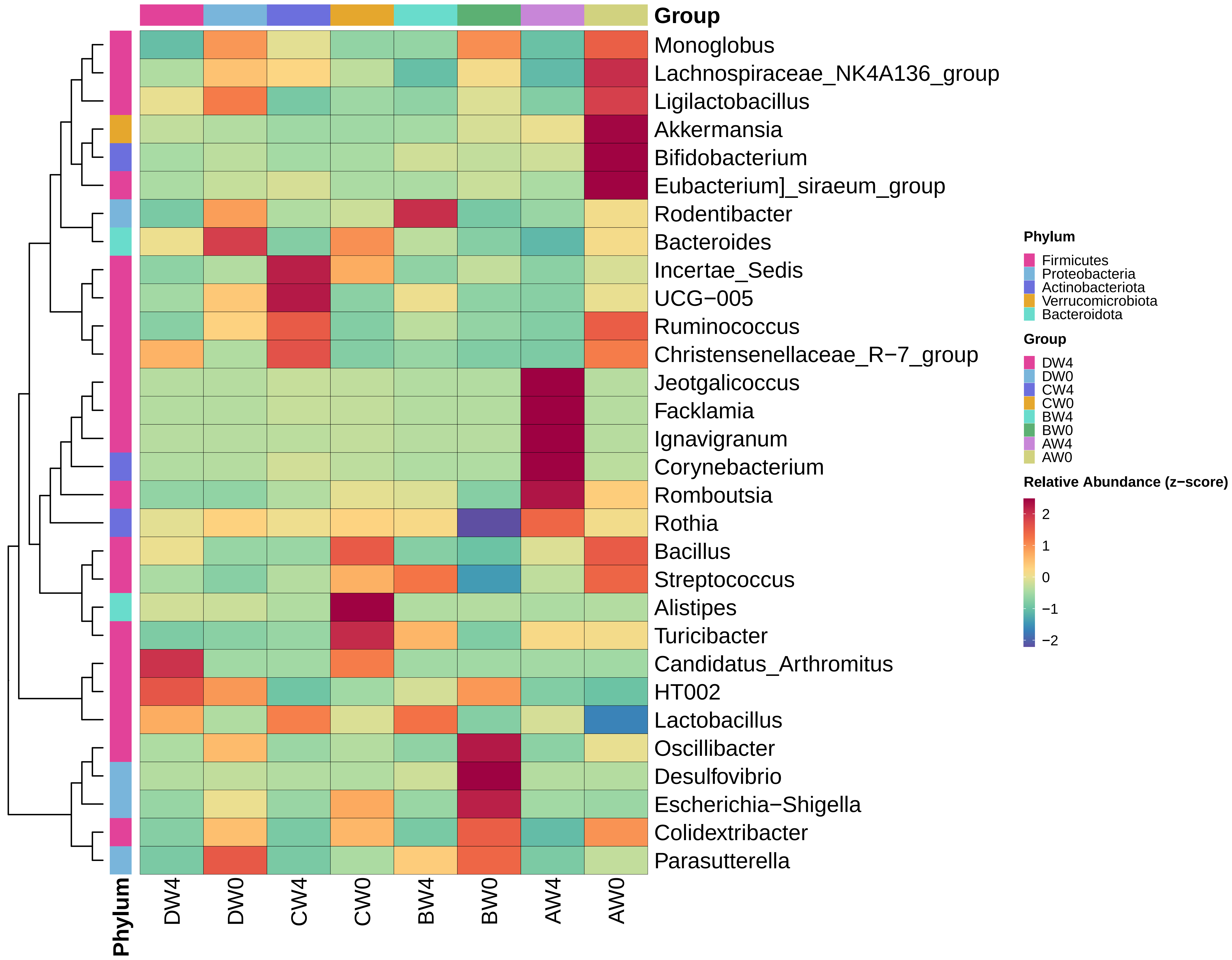

Group
Monoglobus
Lachnospiraceae_NK4A136_group
Ligilactobacillus
Akkermansia
Bifidobacterium
Eubacterium]_siraeum_group
Rodentibacter
Bacteroides
Incertae_Sedis
UCG−005
Ruminococcus
Christensenellaceae_R−7_group
Jeotgalicoccus
Facklamia
Ignavigranum
Corynebacterium
Romboutsia
Rothia
Bacillus
Streptococcus
Alistipes
Turicibacter
Candidatus_Arthromitus
HT002
Lactobacillus
Oscillibacter
Desulfovibrio
Escherichia−Shigella
Colidextribacter
Parasutterella
Phylum
DW4
DW0
CW4
CW0
BW4
BW0
AW4
AW0
Phylum
Firmicutes
Proteobacteria
Actinobacteriota
Verrucomicrobiota
Bacteroidota
Group
DW4
DW0
CW4
CW0
BW4
BW0
AW4
AW0
Relative Abundance (z−score)
2
1
0
−1
−2
